# A Real-Time Physical Progress Measurement Method for Schedule Performance Control Using Vision, an AR Marker and Machine Learning in a Ship Block Assembly Process

**DOI:** 10.3390/s20185386

**Published:** 2020-09-20

**Authors:** Taihun Choi, Yoonho Seo

**Affiliations:** 1 Department of Industrial and Management Engineering, Korea University, Seoul KS013, Korea; taehoon.choi@ksoe.co.kr or; 2 Korea Shipbuilding and Offshore Engineering, Seoul KS013, Korea

**Keywords:** performance measurement, process progress management, AR marker, machine learning, Internet of Things (IoT), smart shipyard, Industry 4.0

## Abstract

Progress control is a key technology for successfully carrying out a project by predicting possible problems, particularly production delays, and establishing measures to avoid them (decision-making). However, shipyard progress management is still dependent on the empirical judgment of the manager, and this has led to delays in delivery, which raises ship production costs. Therefore, this paper proposes a methodology for shipyard ship block assembly plants that enables objective process progress measurement based on real-time work performance data, rather than the empirical judgment of a site manager. In particular, an IoT-based physical progress measurement method that can automatically measure work performance without human intervention is presented for the mounting and welding activities of ship block assembly work. Both an augmented reality (AR) marker-based image analysis system and a welding machine time-series data-based machine learning model are presented for measuring the performances of the mounting and welding activities. In addition, the physical progress measurement method proposed in this study was applied to the ship block assembly plant of shipyard H to verify its validity.

## 1. Introduction

To improve productivity and enhance cost competitiveness, the shipbuilding industry aims to establish smart shipyards that can manage performance through the real-time collection of production information. Progress control is a key technology for successfully carrying out a project by predicting possible problems, such as production delays, and establishing measures to avoid them (decision-making) [1,2,3].

Recently, the shipbuilding industry has become more complex in terms of production site management due to the mixed production practices caused by the diversification of ship types and shapes. As a result, production schedules and executions often fail to match, resulting in too much time and money being needed to identify process status, and complicating the immediate actions taken by managers in response to changes in site conditions. Therefore, a rapid and accurate process performance management system is required to allow appropriate actions in response to changes in site conditions.

However, shipyard performance management is still based on the task manager’s empirical (intuitive) know-how [4,5]. When wrong decisions are made, the reliance of this empirical (intuitive) progress management on the judgment of the production manager increases vessel production costs due to missed vessel production delivery dates. Reasons for relying on the task manager’s empirical know-how are the lack of data reliability and the excessive manpower and costs associated with data collection. Firstly, at shipyards most production work and performance data collection are done by workers, making production data both difficult to collect in real-time and inaccurate. Secondly, much manpower and time is required to measure the work performance related to the numerous ship blocks deployed in the shipbuilding workshop.

The digital twin/cyber physical system (CPS), Internet of Things (IoT), big data, cloud and robots, which have recently emerged as the wave of the fourth industrial revolution (the Industry 4.0), can present new solutions as alternatives to the various problems and existing empirical performance measurement methods at shipyards [6,7,8]. In other words, the foundations of the Industry 4.0 can be transferred straight to a Shipyard 4.0. The deployment of digital twin (or CPS) in production systems creates the “smart factory” similar to the “smart shipyard” [9].

The digital twin is a technology that creates twins (virtual models) of the physical objects on a computer and predicts what may happen in the real world through simulation [10]. The virtual models can analyze the current state through network connectivity with physical objects and predict the dynamic changes. The physical objects receive the optimized operational results from virtual models and reflect them [11]. Through the cyber-physical parallel control, the digital twin can achieve the optimization of the entire manufacturing process [12]. With the integration of products, processes, resources and businesses, the production can operate in a flexible, efficient and eco-friendly way, providing cost advantages in comparison with traditional production systems. Equipment will be able to improve process performance through the autonomous decision making and self-optimization. In the application of the digital twin, the shipbuilding industry faces challenges, such as the vertical integration of production systems, the horizontal integration of value-added networks and the acceleration of digital technologies required for re-engineering the entire value chain [13,14]. In particular, the vertical integration of cyber and physical systems, which can create new added value to the shipyard production chain, is required first. The IoT is a digital technology for vertical integration and can provide a solution to automatically collecting and transmitting data throughout the manufacturing process, reducing the time and costs required to secure production data and measure performance.

Therefore, in order to implement digital twin-based smart shipyards, automated performance measurement methods of physical systems are first required. Studies have recently been conducted into collecting and analyzing data at shipyard production sites in real time and then using that data for production management and performance management.

Chang et al. [15] demonstrated a real-time production information system in a shipyard panel block assembly factory that used a wireless network and a personal digital assistant (PDA) and introduced a manufacturing execution system (MES). Hwang et al. [16] developed a manufacturing execution system (MES) for a panel block assembly line by utilizing mobile devices. In these two studies, considering the poor production environments of many shipyards, mobile devices (such as PDAs) were used for performance data collection. To improve work efficiency, Lee and Kim [17] conducted a study proposing smart work that relied on the usage of smart devices, such as smartphones and tablet PCs, to perform tasks with less time and place limitations. Smart work is expected to enable more efficient production data collection and management of information in shipyards.

Paula Faga-Lamas et al. [13] proposed a smart pipe system that could enhance the productivity of a piping process by applying radio frequency identification (RFID) technology in a shipyard plumbing process to automatically recognize, locate and optimize pipe routing. Noh and Shin [18] designed the monitoring system using RFID for the curved plates forming process in the curved ship-block assembly plant.

Lee [19] described a sensor-based remote monitoring technique for ship block assembly and the implementation of a prototype system. This approach attaches some distance-measuring sensor nodes to ship blocks instead of manual processing. Remote monitoring of the assembly status can minimize marginal errors during the ship block assembly process.

Kim, M. et al. [20] proposed a vision-based monitoring system for the ship-block assembly process. Ship block images acquired from the camera are processed to extract the areas of the ship blocks. The extracted ship blocks are compared with CAD data to estimate the assembly progress. The vision-based monitoring system can automatically estimate the current progress of ship block assembly, thereby improving the entire ship building process. However, the estimation of assembly progress may become inaccurate when components added on the block are not clearly detected.

Blanco-Novoa, O. et al. [21] described the validation of the technologies necessary to design an industrial augmented reality (IAR) system for developing applications for an Industry 4.0 shipyard. The main IAR use cases were described concerning the IAR communication architecture deployed by Navantia Shipyard in Ferrol (Spain). Fernandez-Carames, T.M. et al. [22] described Navantia’s IAR architecture using cloudlets and fog computing to reduce the latency of responses to traditional cloud-based systems. After describing the proposed architecture in detail, the system was evaluated with various real scenarios and different payload sizes. This architecture can provide low-latency responses and may develop real-time IAR applications for the shipyard 4.0.

In terms of project management, performance measurement research has also focused on the construction industry, which is similar to the shipbuilding industry. Golparvar-Fard, M. et al. [23] developed an automated progress measurement method that analyzes and visualizes progress using daily field photographs and 4D IFC-based building information models. Behnam, A. et al. [24] made an online progress reporting and automated progress measurement system for repetitive construction tasks using satellite remote sensing technology in linear infrastructure projects. Mahami, H. et al. [25] presented an automated construction progress monitoring method using site image and building information modeling. The quantity of the work performed is determined by creating an as-built 3D point cloud of the measured target and comparing it with the as-planned model. The proposed method improves the accuracy and completeness of the generated point cloud and provides a more accurate view of actual project progress.

As such, recent studies have been conducted to improve both the convenience and performance of data collection and streamline production management by converging ICT (information and communication technologies) into existing shipyard production processes. However, there is still a lack of research into how real-time performance information can be collected at shipyards without the intervention of workers or objective progress measurement based on production data. Therefore, in this study, an IoT-based performance measurement method is presented that can automatically collect performance data without worker intervention through analyzing the work procedures and facilities used in the ship block assembly process. In addition, a real-time workload-based progress measurement method is proposed that allows objective performance measurement using actual work data collected in real time. Finally, the proposed method was applied to the ship block assembly plant in shipyard H to verify its validity.

## 2. An Analysis of the Ship Block Assembly Process in Shipyards

### 2.1. Work Procedures of the Ship Block Assembly Process

For this study on automatic performance data collection in real time in the assembly process of shipyard ship blocks, the curved block assembly plant was selected as the subject of analysis, and both the work procedure and process facilities were analyzed. Since generally the curved block assembly plant acts as an overall bottleneck in the ship assembly process, and it has a significant impact on the forward and rear processes, and this plant requires very precise performance control. The following describes the general assembly process at the curved block assembly workshop.

**Figure 1:**
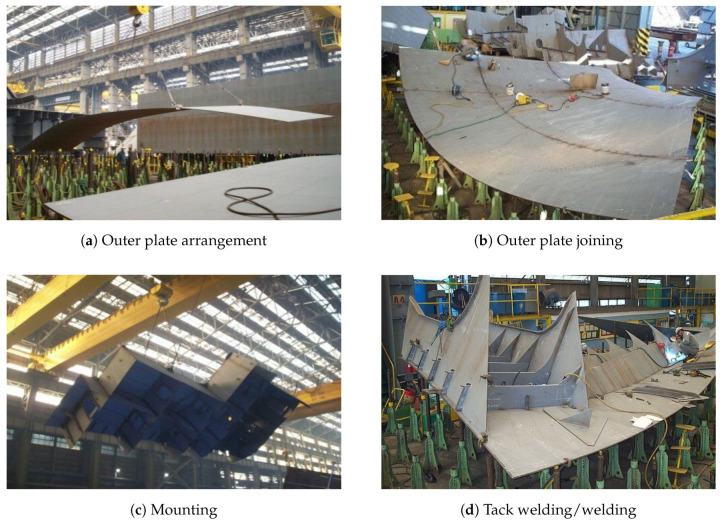
Working activities in the curved block assembly process.

1. PIN JIG setting: Before placing the ship’s outer plates on the jig, the height of the pin jig is adjusted according to the curvature of the ship’s outer plate.
2. Ship’s outer plate arrangement and joining: The ship’s outer plates are arranged on a controlled pin jig and the outer plates are welded. The outer plates are moved by overhead cranes and placed in the workshop. Welding machines are used for joining operations on the outer plates. Figure 1a shows outer plates being placed on top of the pin jig. Figure 1b shows the outer plates being bonded by the welder.
3. Mounting: Various materials and subassembly blocks are put on the ship’s outer plates to produce the final shape of the ship block. Figure 1c shows a subassembly block being moved by a crane to be mounted on the outer plates.
4. Tack welding/welding (main welding): Work pieces, such as materials and subassemblies, placed on the ship’s outer plates are fixed in position by tack welding and then the work pieces are bonded by welding. Generally, tack welding is done before the main welding. Figure 1d shows a subassembly block mounted on the ship’s outer plates being welded by a welder.
5. Grinding: Weld grinding is cleanly removing the excess weld metal. The duration of this grinding activity depends on the quality of welding; good quality welding reduces grinding time.
6. Inspection/transfer: Once the ship block has finished the grinding process, it will be transported to an outdoor waiting area after an inspection. An overhead crane is used to transfer the ship block out of the workshop.

**Figure 2:**
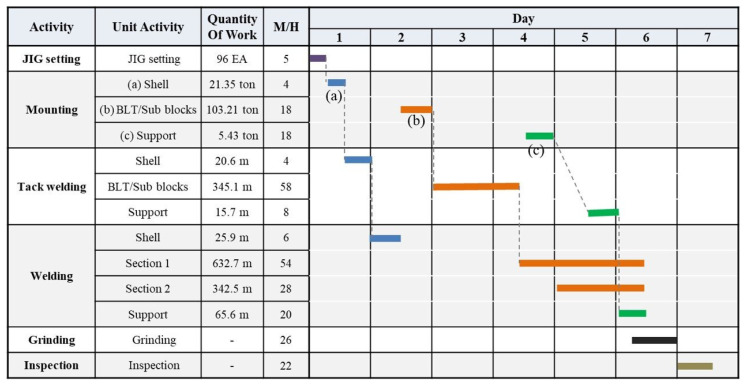
Example of a curved block assembly schedule.

The ship block assembly process described above consists mostly of mounting and welding ship block work pieces. Welding activity management needs to consider two distinct activities, tack welding and welding. Figure 2 shows an example of the ship block assembly schedule. In general, the ship block assembly processes follows sequentially from jig setting to the inspection/transfer operations. However, the mounting and tack welding/welding activities, which involve the highest workloads during the overall process, are performed repeatedly according to the number of components (units of assembled work pieces) in the ship block. In Figure 2, (a) Shell, (b) BLT/Sub blocks and (c) Support show examples of ship block assembly components. Here, mounting, tack welding and welding activities can be seen repeating according to the number of components. Therefore, ship block assembly process performance measurement considers work activity in terms of ship block components rather than the completion of each process. Currently, most shipyards rely on site managers’ empirical judgments and process meetings to measure performance by work activity.

### 2.2. Current Methods for Measuring the Progress of Ship Blocks in a Shipyard

In general, there are three main types of process progress measurement methods: the estimated percent complete method (EPCM), the earned value method (EVM), and the physical progress measurement method (PPMM) [1,2,3].

First, the EPCM is a method by which a control manager of a process or activity identifies the progress of work and assigns a progress rate based on his/her subjective judgment. Progress measurement standards are very simple, advantageous for simple repetitive construction and can save time and man-hours. However, they lack objectivity and are likely to be inconsistent with the relationship between the progress of a process and the actual workload.

Second, the EVM is a method of calculating progress rates by dividing the unit scope into measurable scales and marking achievement progress by the stage of the work. The more detailed the level of segmentation is, the more objective the measure of progress will be.

Third, the PPMM is a method of measuring actual work completed as a percentage of the total planned work volume, allowing meaningful progress rates. Of the three methods of measuring progress, this requires the highest level of objectivity regarding work volume. In practice, measuring the actual workload takes many man-hours and much time.

The generally used means of progress management of a shipyard—the subject of this paper—is EPCM based on the empirical (intuitive) judgment of the field manager. This is because the assembly work of a shipyard’s ship block is difficult to break down into measurable levels of work and the manpower and time needed to measure the actual workload are lacking.

**Figure 3:**
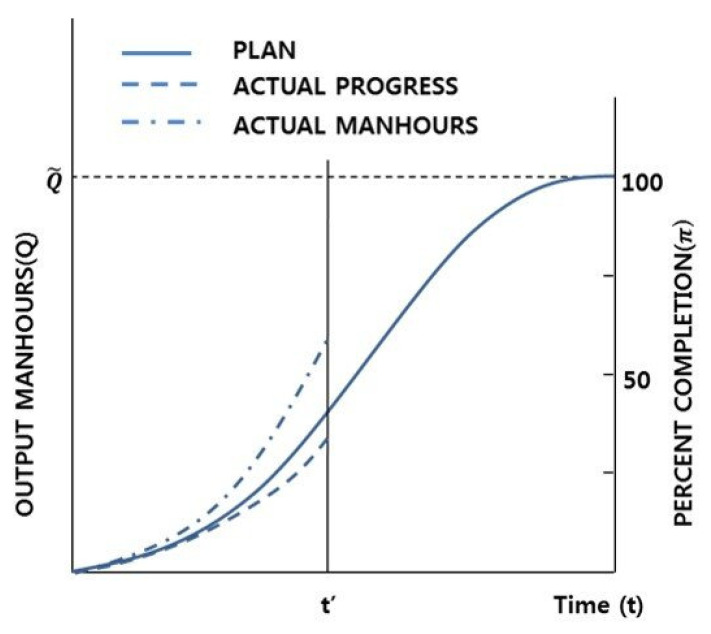
Sample ship block production progress curve.

Figure 3 shows an example of the production progress curve of a ship block [26]. For this example, assume that the planned progress rate, the actual physical progress rate and the total budget actually used are 40%, 35% and 60%, respectively, at the time t’. At this point, the progress of the plan and the actual budget used can be known objectively. However, because actual physical progress is empirically estimated, it can be determined differently by different field managers. For example, if the field manager estimates that the physical progress is 60%, the same as the proportion of the budget used, the progress through the schedule is judged to be ahead of the planned schedule. However, the actual physical progress is behind the planned schedule. These errors are common in shipyards and can cause delays in the process.

Therefore, achieving the objective progress measurement and reliable decision-making required by the shipyard requires not only a real-time work performance collection system that can reduce the time and manpower required for collecting work information but also a new method for objective progress measurement based on actual workload.

## 3. Proposal of a Progress Measurement Method for the Ship Block Assembly Process

The ship block assembly process consists of the mounting, tack welding, welding, grinding and inspection activities described in Section 2.1, and each is managed separately. Therefore, in this study, methods of measuring performance are presented for each work activity in the shipyard block assembly process. Then, a method is presented to automatically measure the actual workload of the ship block by applying Internet of Things technology.

The total progress of the ship block *j* ($TP_{j}$) to be measured in this study can be defined as the sum of the progress of activity *i* ($PA_{ij}$) generated in the ship block *j*. In this case, activity *i* includes the preparation of the operation (such as the jig setting), the mounting, the tack welding, the welding, the grinding and the inspection. Here, the working ratio of activity *i* in the assembly process of the ship block is different. The completion of activity *i* is seen as a percentage of work done by activity *i* in the entire ship block assembly process. Thus, the work-weight constant $c_{ij}$ is defined as the amount of work occupied by activity *i* in the total workload of ship block *j*. $c_{ij}$ varies depending on the type of ship block and is calculated from the standard budget for activity *i* of ship block *j*. The total progress of the ship block is shown in Equation (1):

(1) $$TP_{j}\;=\;\sum_{i=1}^{n}\;\left(\;C_{ij}\;\times \;PA_{ij}\;\right)$$

In Equation (2), the method of measuring the progress of the activity *i* ($PA_{ij}$) must be defined in order to calculate the total progress rate ($TP_{j}$) of ship block *j*. This study applies PPMM, which allows the measurement of performance in terms of overall workload, to mounting and welding activities, which, of all the activities *i* of ship block assembly, require particularly precise progress management. EVM is proposed for the jig setting, grinding and inspection parts of activity *i* for which only management of the completion of the work is required. The reason why PPMM is used to measure the progress of the mounting and welding activities is that the mounting and welding work generally takes up 80% or more of the ship block work and since the duration of the work is long it is very important to measure performance based on actual workload. The progress measurement formula for the mounting, tack welding and welding of activity *i* is shown in Equation (2). $TW_{ij}$ is the total work volume of activity *i* in ship block *j*. $AW_{ij}$ is the actual amount of work completed during the operation of activity *i* in ship block *j*.

(2) $$\begin{array}{ccc} PA_{ij}\; & = & AW_{ij}/TW_{ij}\;,\\ & & when\;activity\;i\;=\;Mounting,\;Tact\;welding,\;Welding \end{array}$$

The progress measurement formula for JIG setting, grinding and inspection among activity *i* is shown in Equation (3).

(3) $$\begin{array}{ccc} PA_{ij}\; & = & \;1\;or\;0\;,\\ & & when\;activity\;i\;=\;JIG\;setting,\;Grinding,\;Inspection \end{array}$$

Table 1 shows an example of ship block progress measurement, as measured by the method presented in this study. Table 1 shows that the JIG setting activity has been completed, and that the mounting, tack welding and welding are in progress at 14.4%, 15.0% and 12.5% respectively for the entire operation. The grinding activity is managed by subdividing it into Section 1 and Section 2.

Methods for measuring the performance of the mounting and welding activities are subject to physical progress measurement in this study are described in Section 4 and Section 5 of the study. Section 6 of the study describes the results of applying the physical progress measurement method of this study to ship block assembly workshop in the H shipyard.

## 4. Automated Progress Measurement of Mounting Activity Using Vision and Marker

As described in Section 2.1, the mounting activity is the placement of various materials and subassembly blocks on ship block outer plates for assembly in the final shape of the ship block. It takes much time and manpower to measure the progress of the mounting activity of the numerous ship blocks deployed in the shipyard’s workshops.

Recently, imaging systems have been used as work performance measurement methods in various industries in order to reduce time and manpower required for performance data collection [25,27,28]. The method of measuring process progress using the imaging system is to measure progress by comparing as-planned models and real time as-built images collected at the site. However, since the ship block assembly plant has low illumination and most of the workpieces and ship blocks are a series of gray colors, it is very difficult to measure the progress of the process by analyzing the images of the ship blocks.

**Figure 4:**
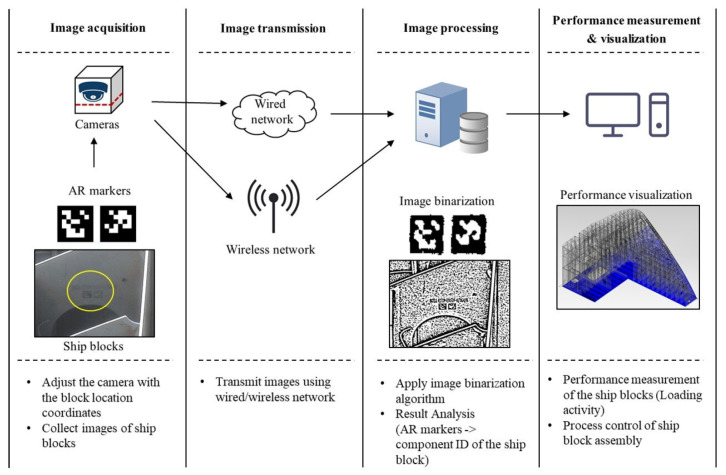
Procedure of the performance measurement of mounting activity.

Thus, in this study, an imaging analysis method using a marker is proposed to facilitate analysis of the process progress of ship block mounting activities. The method of image analysis using a marker proposed in this study consists of four steps, as shown in Figure 4.

### 4.1. Image Acquisition (AR Markers)

Augmented reality (AR) markers are visual signals which trigger the display of the virtual information [21]. Generally, markers are symbols that help recognize objects, and they include QR codes and AR markers [29,30,31,32,33]. AR markers have the advantage of high recognition rates because their shapes are simpler than QR codes. In this study, AR markers were used to recognize components mounted on ship blocks.

Using the camera installed on-site, ship block images were collected and the AR markers in images were analyzed to recognize the components mounted on the ship block. AR markers were printed on steel plates in the preprocessing process of the shipyard.

**Figure 5:**
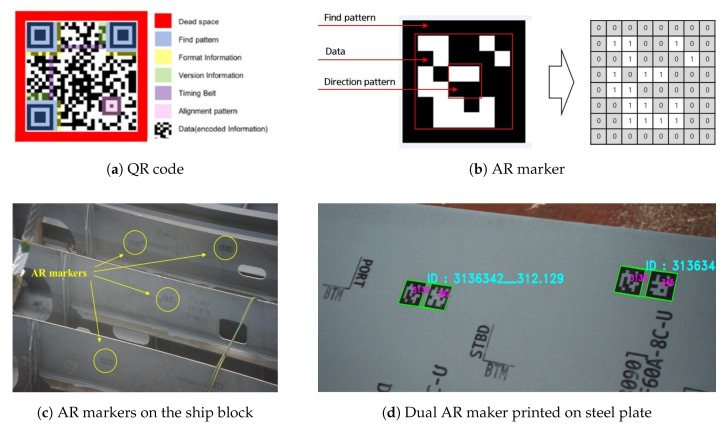
QR code, AR maker, AR markers on the ship block and dual AR maker printed on steel plate.

**Figure 6:**
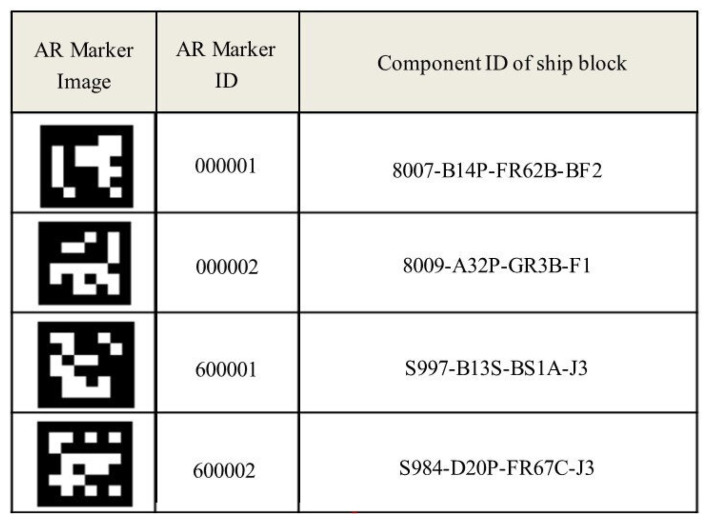
An example of the mapping of the AR marker IDs and ship block component IDs.

Figure 5 shows examples of a QR code, AR markers, AR markers on the ship block and AR markers printed on steel plates, respectively. The AR marker used in this study consists of a two-dimensional bit pattern of size 6 × 6 and a black border area surrounding it. The black border area (find pattern) is intended to allow quick marker recognition, and the internal two-dimensional bit pattern (data) expresses the unique ID of each AR marker through a combination of white and black cells [34]. An 8 × 8 AR marker (including a border) can present an integer from 000 to 999. In this study, dual AR markers were used to express the numerous material IDs required for the production of ship blocks. The use of dual AR markers allows unique ID representations from 000000 to 999999. In the case of the ship block assembly factory of shipyard H, the number of components to be marked does not exceed 400,000, even considering all the blocks placed in the workshop. Moreover, once the block is completed and taken out of the workshop, the AR marker IDs can be reused. Figure 6 illustrates an example of the mapping of the AR marker IDs and ship block component IDs. For AR Marker IDs, sub-materials were allocated from 000001 to 600000, and steel plates were allocated from 600001 to 99999.

**Figure 7:**
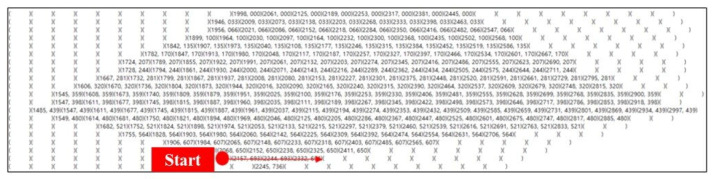
Setting up image-taking area coordinates for the ship block workshop.

The images of the ship block were taken sequentially in an S pattern from the lower left corner of the workshop based on preset coordinate values. Figure 7 shows the coordinate values of the areas where imaging was required in the workshop. The "x" mark indicates an area where no image was required due to an obstacle.

### 4.2. Image Transmission

Ship block images collected at the site are transmitted to the server using a wired/wireless network. More than 200 images are created for a ship block. However, the quantity of images collected depends on the size of the ship block and workshop.

### 4.3. Image Processing and AR Marker Analysis

To simplify image analysis, the collected AR marker images need to be converted to images consisting of only black and white pixels from those consisting of colored or gray pixels. This pre-processing of images is called “image binarization”. In this study, the adaptive thresholding method was used for image binarization [35,36]. Adaptive thresholding is more useful than thresholding for site situations where the brightness is not uniform.

The AR marker images collected in this study had unclear marker boundaries and contained much noise due to the low light environment of the site. Thus, the images had to undergo a smoothing process before binarization. This study used the Gaussian blur method and the bilateral filter method for image smoothing [37,38,39,40]. The Gaussian blur method is most effective for eliminating Gaussian noise (white noise of uniform density) from the image. Bilateral filtering is a method of Gaussian blurring while maintaining boundaries.

**Figure 8:**
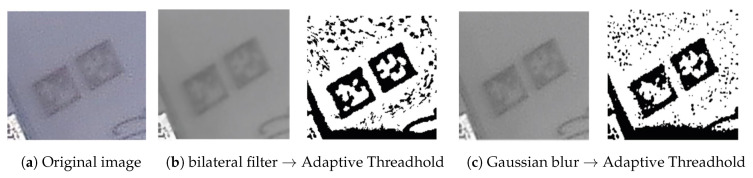
Image binarization.

**Figure 9:**
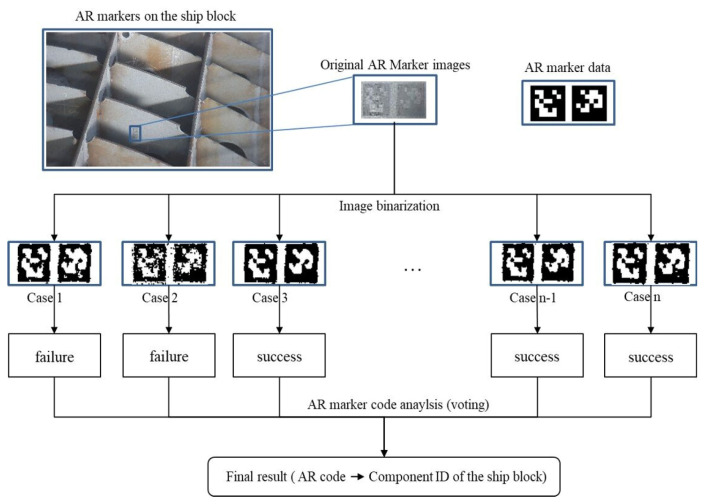
Procedure for AR marker analysis.

Figure 8 shows an example of binarization using the Gaussian blur method and the bilateral filter method. In the results of the binarizations using the Gaussian blur method and bilateral filter method, respectively, the bilateral filter showed better performance at the ship block assembly site. However, because the test results were complementary to each other, this study generated 12 binarization cases (Gaussian blur method (6), bilateral filter method (6)) using both algorithms for AR marker analysis, and then merged the analysis results to from the final result. The binarization cases appropriate for the illumination conditions of the site were determined. Figure 9 illustrates the procedure for analyzing AR markers.

### 4.4. Performance Measurement and Visualization

The analyzed AR marker is mapped to the unique ID of the ship block material. Therefore, comparing the assembly diagram and AR markers of the ship block shows the current process progress of the ship block being worked on. Here, since the unique ID of the AR marker is the material ID on the assembly diagram of the ship block, if any of the AR markers are detected, it is assumed that the entire component (one intermediate assembly on the assembly diagram) is mounted. One component of a ship block is made of several materials.

The progress of the mounting activity ($PA_{ij}$) is measured by calculating the sum of the weights of the mounted workpieces k in ship block *j* ($MWB_{jk})$ and dividing that by the total weight of the ship block *j* ($TWB_{j}$). At this time, the actual mounted workpiece k can be identified by analyzing the collected AR markers. The progress of the mounting activity of ship block *j* is as shown in Equation (4).

(4) $$\begin{array}{cc} PA_{ij}\; & =\frac{\sum_{k=1}^{n}MWB_{jk}}{TWB_{j}}\;,\;when\;activity\;i\;=\;Mounting \end{array}$$

**Figure 10:**
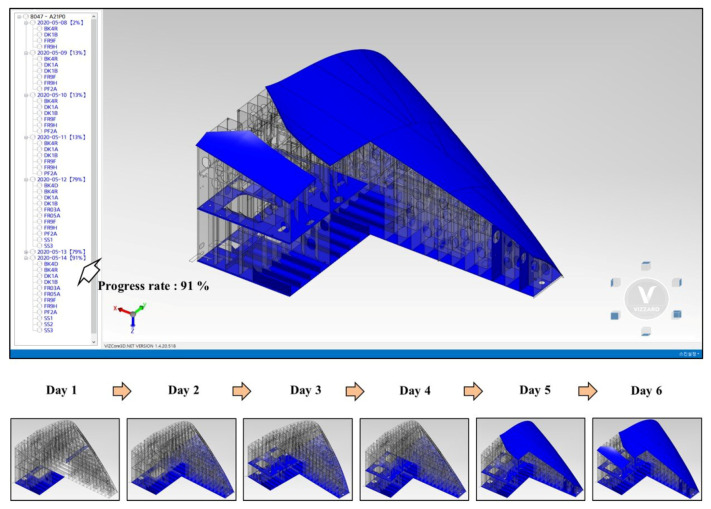
Visualization of mounting activity.

Figure 10 shows the mounted state of the ship block by cross-referencing the ship block components recognized by AR marker with the design drawings. It also shows the status of the ship blocks mounted by date. The ship block in Figure 10 has been worked on for six days and has a 91% progress rate. This system was developed with .Net Framework 4.6 (Micorsoft) and VIZCore3D.NET (Softhills).

## 5. Automated Progress Measurement of Weld Activity Using Weld Sensor Data

**Figure 11:**
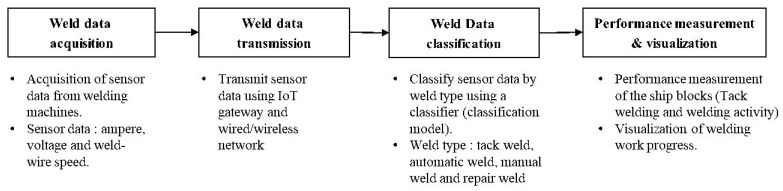
Procedure for measuring the progress of welding activity.

This chapter proposes a method for automatically measuring the performance of welding work using sensor data, particularly that of current, voltage and weld-wire speed, generated by welders. As in the ship block assembly process tack welding and welding account for more than 60% of the total workload and are time-consuming, it is very important to objectively manage the performances of welding activities. Tack welding and welding are done by different workers. Available budgets are also managed separately. Thus, this study proposes a machine learning-based welding type classification model that can automatically classify tack welding and welding work through supervised learning from sensor data. The proposed method consists of the following procedures shown in Figure 11.

### 5.1. Acquisition and Transmission of Welding Data

**Figure 12:**
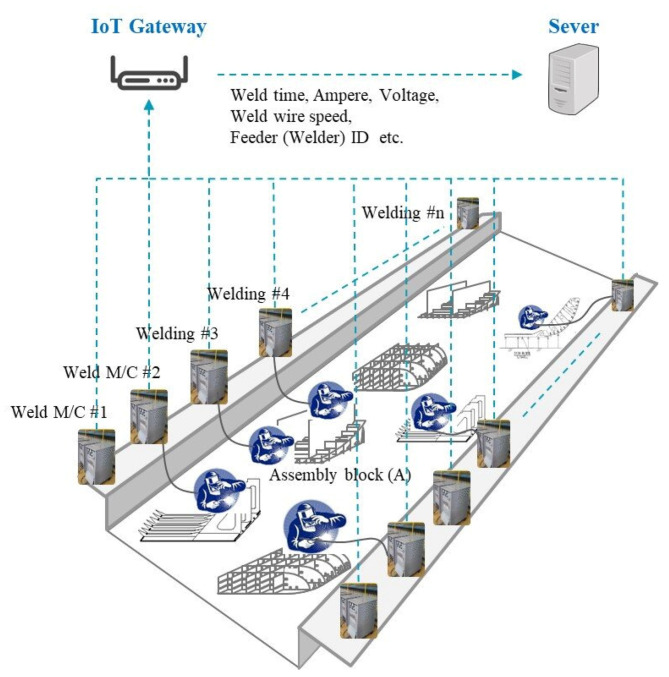
Configuration diagram of the welding data acquisition system.

In this study, to collect the working data of the welder, an electric current sensor and voltage sensor and a welding wire feed speed sensor were attached to welding machines and a unique ID was given to the weld feeder. After collecting the time series welding data from each sensor, the data are sent to an IoT gateway. The IoT gateway combines the received welding machine sensor data and time data and sends them to an analysis server. The data are transmitted every second. Figure 12 shows the system configuration for collecting welding data at the shipyard block assembly site. For example, at the assembly block (A) in Figure 12, two welders are welding using welding machines 3 and 4. To calculate each workload the welding data from work underway by the welder are collected in real time from the welding machines and automatically classified into tack welding or welding through a machine learning algorithm.

### 5.2. Machine Learning-Based Welding Type Classification Model

This section presents the welding type classification model and evaluates its performance at automatically distinguishing sensor data collected from welding machines doing tag welding or normal welding.

#### 5.2.1. Visualization and Data Processing of Welding Data

Using real-time welding time series data, the proposed method of measuring sensor-data-based tack welding/welding activity performance predicts welding types and calculates the performance of the predicted welding work. To predict the welding type from the welding time-series data (current, voltage and weld-wire feeding speed) collected from the welding machines, first the respective features of each type of welding operation need to be identified in the time series data. In ship block assembly work, the types of welding include tag welding, welding (automatic or manual) and repair welding. Automatic welding is welding using an automatic welding carriage machine, and manual welding is welding done manually by an operator using a welding torch.

**Figure 13:**
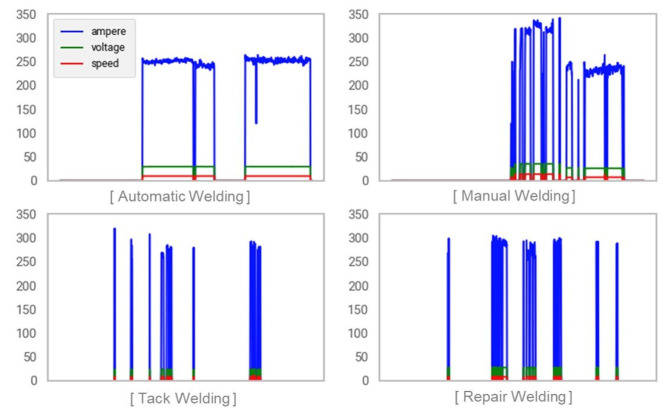
Time series graph by welding operation.

In this study, the electric current (ampere), voltage and weld-wire feed speed of each welding operation were visualized, as shown in Figure 13, in order to extract features of the sensor data corresponding to the different types of welding work. In ship block assembly work, the types of welding include tack welding, welding (automatic, manual) and repair welding. Repair welding is not measured as work performance because it is performed on weld defects. Automatic welding is welding using an automatic welding carriage machine, and manual welding is welding done manually by an operator using a welding torch.

Looking closely at the time series graph for each type of work, automatic welding has the characteristics of long continuous working hours and small variations in sensor data. Tack welding and repair welding have short continuous working hours and large variations in sensor data. Manual welding has a complex mix of characteristics found for automatic welding, tack welding and repair welding, has some continuous working time and has a large variation in sensor data.

**Figure 14:**
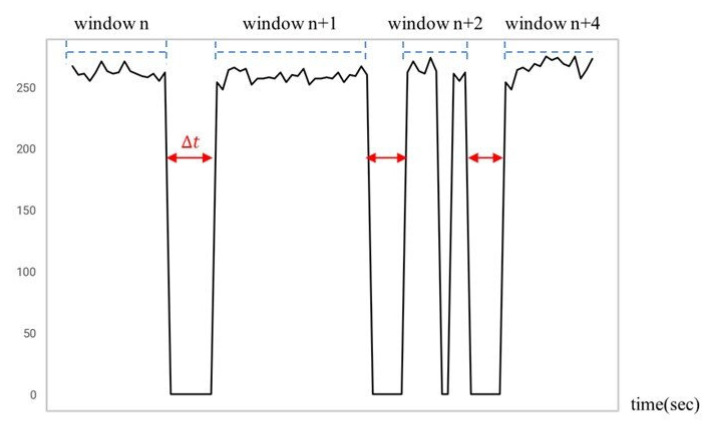
Session windowing for the weld time-series data.

In this study, session window techniques were used to transform the time series data collected from welding machines into datasets suitable for supervised learning. Windowing refers to the way in which time series data are processed in real time and windowing techniques include tumbling (or fixed) windows, hopping (or sliding) windows and session windows [41,42,43]. Figure 14 illustrates the processing of time-series data with the session window method. The reason for using session window techniques in this study is that welding time series data have independent data intervals. In this study, for real-time analysis of weld time series data, the collected data were delineated as a single window when Δt > the inactivity gap threshold of 60 s.

#### 5.2.2. Feature Extraction and Classification Models

To implement the welding type classification model through supervised learning, sensor data from welding machines and welding work type data are required, where the sensor time series data are used as the independent variables for the model, and the welding operation type data are used as the dependent variables. In this study, the time series graph shows that features such as sensor data value variation over time or continuous working time are important factors for distinguishing between the different types of welding work. Thus, the statistical information of the sensor data is used as an independent variable for the welding type classification model. First, as shown in Equation (5), the average current $a_{i}$, average voltage $v_{i}$ and average welding wire feed speed $s_{i}$ were calculated. $td_{i}$ is the working time of a continuous operation and *i* is the work number.

(5) $$\begin{array}{c} a_{i},\;v_{i},\;s_{i}\;=\frac{\sum_{i=1}^{n}\sum_{j=td_{i-1}+1}^{td_{i}}a_{j},\;v_{j},\;s_{j}}{td_{i}},\;td_{0}=0 \end{array}$$

In order to calculate the variation in sensor data during continuous working hours, the current variance $va_{i}$ voltage variance $vv_{i}$, and welding wire feed speed variance $vs_{i}$ during $td_{i}$ were calculated as shown in Equation (6).

(6) $$\begin{array}{c} va_{i},\;vv_{i},\;vs_{i}\;=\frac{\sum_{i=0}^{n}\sum_{j=td_{i-1}+1}^{td_{i}}{\left(a_{j},\;v_{j},\;s_{j}\;-\;a_{i},\;v_{i},\;s_{i}\right)}^{2}}{td_{i}},\;\;td_{0}=0 \end{array}$$

The variable $cs_{i}$ was defined to increase the degree to which the features of working time continuity were reflected in the model according to the type of welding work. $cs_{i}$ is a variable that indicates the continuity of working hours. variable $cs_{i}$ reflects the larger constant as the continuous working time is longer. It is calculated by Equation (7).

(7) $$\begin{array}{c} cs_{i}\;=\frac{td_{i}\left(td_{i}+1\right)}{2},\;td_{0}=0 \end{array}$$

**Figure 15:**
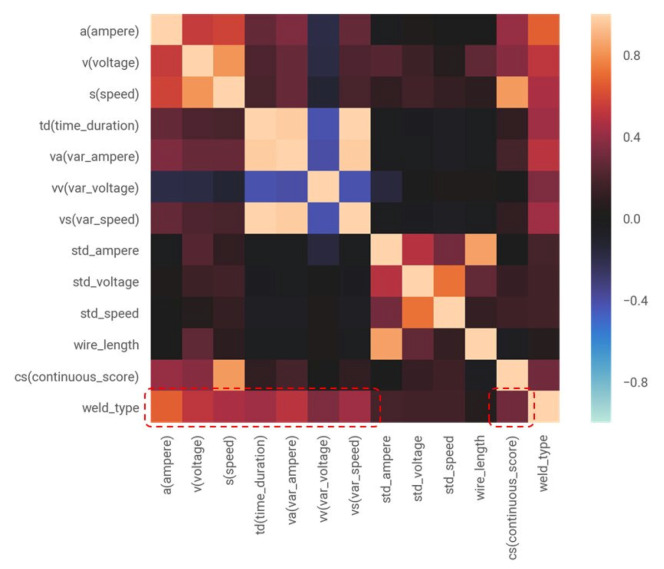
The correlation matrix between feature variables.

In addition, the current standard deviation, the voltage standard deviation, the weld-wire feed speed standard deviation and weld-wire length were extracted as feature variables. Next, to define the feature variables to be used for the weld type classification models, the correlation between the feature variables and the welding types were identified. Figure 15 illustrates the correlation matrix describing the correlations between the feature variables and the welding types.

In Figure 15, the feature variables average current $a_{i}$, average voltage $v_{i}$, average welding wire feed speed $s_{i}$, current variance $va_{i}$, voltage variance $vv_{i}$, welding wire feed speed variance $vs_{i}$ and working time of a continuous operation $td_{i}$ are relatively highly correlated (correlation coefficient > 0.4) with the type of welding. The variable $cs_{i}$ is also considered to be somewhat correlated with the type of welding and included in the feature variable. Conversely, the standard deviations of current, voltage and welding material transmission speed and the length of the weld-wire used during $td_{i}$ can be seen to be weakly correlated with welding type. Therefore, these variables were excluded from the feature variables. In this study, for the supervised learning of weld-type classification models (or classifiers), eight variables, including the average and variance of current, voltage and weld-wire feed speed, the variable $td_{i}$ and the variable $cs_{i}$ were defined as feature vectors $x_{i}^{feature}$. Here, $x_{i}^{feature}$ represents feature vectors corresponding to the characteristics of window i of the time-series weld data. The weld type to be predicted from the weld time series data is defined as the target variable $y_{i}^{target}$. Here, $y_{i}^{target}$ is the weld type of each window i of the time-series weld data. In addition, the training datasets $D_{i}$, for the learning modeling of the classifier were defined. $D_{i}$ is the training dataset in the window *i* for learning the weld type classification model. The formulas of the feature vector $x_{i}^{feature}$, target variable $y_{i}^{target}$ and training datasets $D_{i}$, for weld type classification model are shown in Equations (8)–(10).

(8) $$x_{i}^{feature}\;=\;\left[\;a_{i}\;v_{i}\;s_{i}\;va_{i}\;vv_{i}\;vs_{i}\;td_{i}\;cs_{i}\;\right],$$

(9) $$y_{i}^{taget}\;=k,\;\left(k=1\;\left(Automatic\right),\;2\;\left(Manual\right),\;3\left(Tack\right),\;4\;\left(Repair\right)\right),$$

(10) $$D_{i}\;=\;\left[\;x_{i}^{feature},\;y_{i}^{target}\;\right]$$

#### 5.2.3. Performance Evaluation of Classification Model

To verify the classification model, its performance is evaluated by calculating its precision, recall, F1-score and accuracy through the confusion matrix. A confusion matrix is a table that compares actual values with the results of a classifier, and one is often used to describe the performance of a classification model (or “classifier”) [44]. Table 2 shows an example of a confusion matrix with the classification values true and false.

In Table 2, TP represents the true positive situation when the actual value is true, and the result of the classification model is also true, so the predicted value and the actual value are the same. Conversely, FP represents the false positive situation when the classification model incorrectly predicted false when the actual value was true. FN indicates the false negative situation in which the classification model incorrectly predicted true when the actual value was false. Finally, TN represents a true negative when the actual value is false and the result of the classification model is also a false, and so the actual value and the predicted value match. Equation (11) through (14) represents some performance indicators of the predictive model derived from the confusion matrix [45,46].

(11) $$Precision=\frac{TP}{TP+FP}$$

(12) $$Recall=\frac{TP}{TP+FN}$$

(13) $$Accuracy=\frac{TP+TN}{TP+FP+FN+TN}$$

(14) $$F1-score=2\times \frac{Precision\times Recall}{Precision+Recall}$$

Accuracy above is the evaluation indicator that can most intuitively represent the performance of the model. It indicates the probability that the results of the classification model will match the actual value. The *F*1 score (F-score or F-measurement) is a measure of the accuracy of the test as a harmonized average of precision and recall [45,46]. The *F*1-score is also used to evaluate the performance of a model when the data label is unbalanced.

#### 5.2.4. Experimentation and the Results of the Classification Model

**Figure 16:**
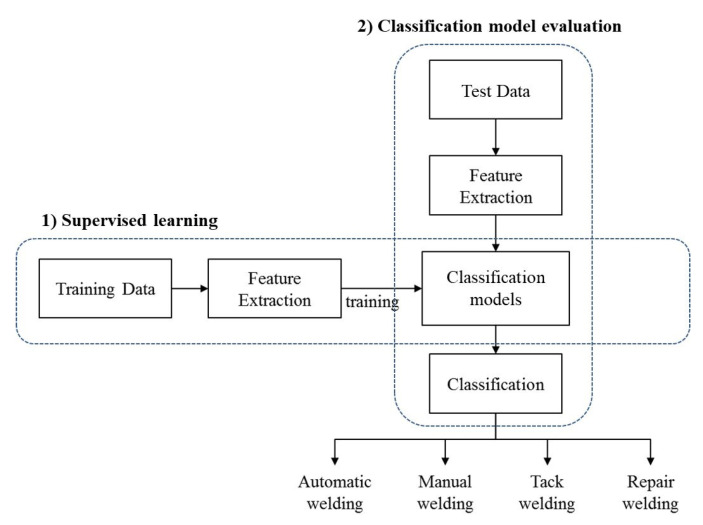
Experimental structure of welding type classification models.

In this subsection, the weld type classifiers are tested and evaluated using actual welding data from shipyard H block assembly plant. The experimental procedure consists of two steps. First, (1) use the training datasets to learn the welding type classification model. Then, (2) enter the test datasets into the welding type classification model to evaluate the performance of the classification models. Figure 16 illustrates the experimental structure diagram of the welding type classification model implementation.

About six months of time series data collected from welding machines at the shipyard H (block assembly plant) were used as data for experimentation and verification. Four months of the data were used as a training dataset and two months as a test dataset. Table 3 shows the number of training and test data sets.

In this study, the performance of the model was compared and evaluated using the ensemble method, which has been used most often in recent machine learning-based classification models as a classifier of the welding type classification model [47,48]. The classifiers used in this study were modeled using the random forest (RF) and extra tree (ET) classifiers in the bagging method, and the gradient boosting (GB), ADA boosting (ADA) and XGBost (XGB) classifiers in the boosting method.

Next, to verify the performances of the five case models selected in this study, a set of test data was entered into the model and the results were written in a confusion matrix. Comparing the actual data by welding type with the predicted results by classification models reveals that automatic welding and manual welding are relatively well classified, but that some data are misclassified for cases of tack welding and repair welding. However, each model has different performance advantages for predicting welding type, so it can be supplemented.

Thus, in this study, stacking model (STK) was also implemented to achieve higher recognition performance and robustness than ultimately utilizing the results of the individual models [49]. The stacking model in this paper used RF as the meta leader model. The sub model also used the RF, ET and GB models to utilize the classification results in the sub model as learning data for the meta leader model. Table 4, Table 5, Table 6, Table 7, Table 8 and Table 9 show the confusion matrices for the six case models.

The performance of the classifier calculated using the confusion matrix is shown in Table 10. Here, the respective accuracies of RF, ET, GB, ADA and XGB were 84.96%, 85.38%, 80.30%, 46.79% and 76.88%. Of the models implemented in this study, the results of the stacking model showed the best perceived performance at 85.43%. However, when looking at its F1-score, its classification performance for automatic and manual welding was high, while its classification performance for tack welding and repair welding was low. Its low classification performance for tack welding and repair welding was judged to be due to their very similar time series data characteristics. Therefore, it is necessary to find new feature variables to improve the classification accuracy for tack welding and repair welding. However, since repair welding is not counted as work performed towards completion of the ship block assembly process, even if repair welding classification performance is low, the welding type classification model presented in this study is considered valid. Table 10 shows the results of evaluating the performance of the classifiers implemented in this study.

### 5.3. Measurement and Visualization of the Classification Performance for Tack-Welding/Normal Welding Activities

To measure the workload of tack-welding/welding in ship block *j*, it is necessary to calculate the workload of the tack-welding/welding of each welder *l* working in ship block *j*. Therefore, the process progress of the tack welding/welding activity ($PA_{ij}$) can be calculated by dividing the sum of the actual welding workload of welder *l* performed in ship block *j* ($AWB_{jl}$) by the total welding workload of ship block *j* ($TWM_{j}$). The process progress of the tack-welding/welding activity of the ship block is shown in Equation (15).

(15) $$\begin{array}{cc} \;PA_{ij}\; & =\frac{\sum_{l=1}^{n}AWB_{jl}}{TWM_{j}}\;,\;when\;activity\;i\;=\;tack\;welding,\;welding \end{array}$$

**Figure 17:**
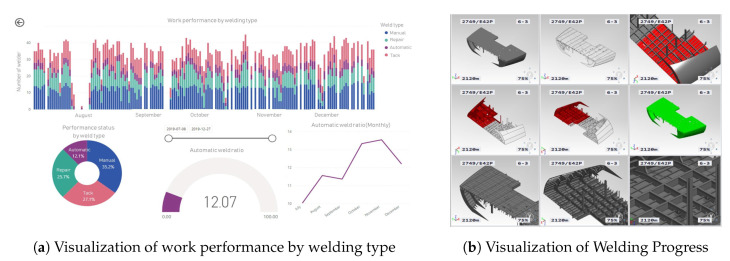
Measurement and visualization of welding activity performance.

Figure 17 shows a visualization of performed welding work activity produced by the welding work classification model proposed in this study. In Figure 17a, the analysis of welding activity over time reveals that tack welding represents 27.1% and welding (automatic, manual) 47.3% of the total. Figure 17b visualizes the welding progress of a ship block.

## 6. Validation

In order to validate the method proposed by this study, the schedule variance of the workplace with and without the proposed method (system) was compared and evaluated over the same period. Four work teams were selected and compared at each workshop. Schedule variance (SV) is the difference between the planned value (PV) and the earned value (EV) [50]. SV < 0 means the process is delayed, while SV > 0 means that the process was completed before its planned completion schedule. In the case of shipyards, when SV = 0, the waiting time of the ship block in the stockyard is minimized, so that can be considered the optimum result.

**Figure 18:**
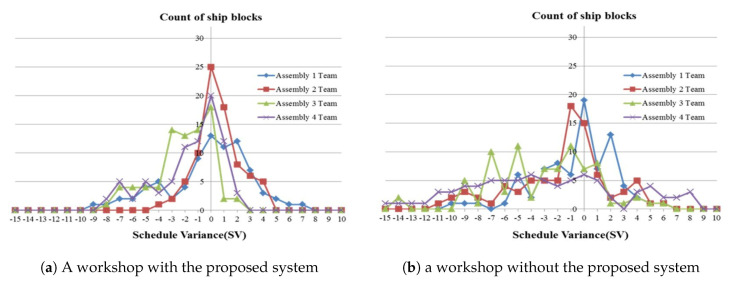
Graph of schedule variance.

**Figure 19:**
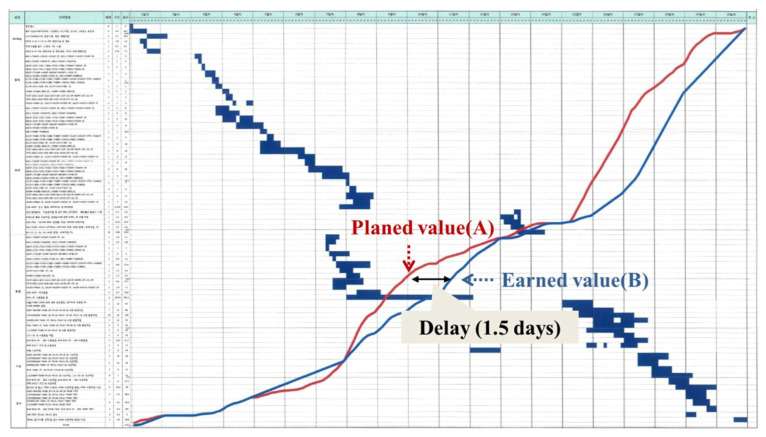
An example of a ship block progress graph.

Figure 18 shows the schedule variance of the ship blocks at the day of completion. Figure 18a shows the schedule variance by ship block of the four work teams in the workshop applying the system proposed in this paper. Figure 18a shows that the ship blocks are clustered around SV = 0, and that the mean SV = −0.86 (days). Figure 18b shows the schedule variance of the ship blocks of four work teams at another workshop without the system applied. Figure 18b shows that the ship blocks are more widely spread out from SV = 0, and that the mean SV = −2.07 (days). On the basis of work team ship block completion time, the schedule variance shown Figure 18a applying the proposed method is smaller and shows a better process control state than the schedule variance without application of the proposed method shown in Figure 18b. This difference can be interpreted as being a result of the PPMM proposed in this study allowing the continuous monitoring of the schedule and rapid process recovery by the site manager in response to delay. For example, Figure 19 shows a graph of the progress of the ship block. There is a 1.5-day delay apparent at point (B) in time, but the site manager can prevent the potential delivery delay in advance by establishing a process recovery plan.

From these results it can be said that the work teams that actively performed process control through the methods presented in this study have excellent schedule compliance.

## 7. Conclusions and Future Research

In this paper, a methodology that enables objective process progress measurement based on data, rather than the empirical judgment of a site manager, was proposed for shipyard ship block assembly plants. To this end, a method was defined for the measurement of work progress by activity that allows for the measurement of overall ship block progress performance. In particular, IoT-based performance collection methods were proposed for mounting and welding activities, which account for most of the work in ship block assembly, and those methods were found capable of automatically measuring actual work volume without human intervention. An AR marker-based image analysis system was proposed as an automatic performance collection method for mounting activity. As part of the method of collecting welding performance activity, a machine learning-based performance measurement method that can analyze performance using the sensor data of a welding machine was presented. In addition, the proposed method was applied to the ship block assembly plant at H Shipyard to verify its validity.

The method presented in this paper means that the collection and processing of on-site shipyard information can be automated, thereby reducing the time and manpower required for the collection of production information and enabling objective and precise performance management. Therefore, the workload-based progress measurement method presented in this paper makes the following contributions to ship block process management. First, it is expected to improve the capability of process management by using PPMM instead of the EPCM by site managers currently used in the shipyard ship block assembly process. Second, by presenting a way to collect actual working data in a shipyard environment directly by utilizing IoT technology, the manpower required for performance collection can be reduced, and the accuracy of the performance information collected can be improved. Third, it is expected that various analysis services based on real-time production data will become available through vertical integration through the physical world and the cyber world, accelerating the implementation of smart shipyards. In addition to this study, more process innovation research applying IoT, big data and AI technologies to shipbuilding will be needed to strengthen the competitiveness of the industry and improve productivity.

In future research, we would like to conduct a study of dynamic scheduling operating on the basis of the work performance automatically collected in this study. Scheduling is a very important area of research for improving production and strengthening shipyard competitiveness. Accordingly, many studies related to scheduling have been carried out by various institutions. Nevertheless, those studies have been mainly conducted on static scheduling because they were based on the assumption that performance was adequately reflected or entered at the time the manager made the plan. However, since this study shows how performance data at the production site can be automatically collected in real time, it is possible to study the dynamic scheduling of changes in real-time production sites. Moreover, it is expected that integration with the results presented in this study will realize the completion of CPS for shipyard assembly block plants.

## Figures and Tables

**Table 1:** Example of progress measurement of ship block *j*.

Activity *i*	Unit	Weight Value (A)	Work Amount or Complete (B)	Progress Rate Activity(%) (A × B)	Performance Mesaurement Method
JIG setting	complete	0.02	1 (Yes)	0.02	EVM
Mounting	ton	0.15	124.56/130	14.4	PPMM
Tack welding	m	0.20	150/200	15	PPMM
Welding	m	0.45	180/650	12.5	PPMM
Grinding (Zone 1)	complete	0.05	0 (No)	0.0	EVM
Grinding (Zone 2)	complete	0.08	0 (No)	0.0	EVM
Total progress rate of ship block *j*				41.9	

**Table 2:** An example of a confusion matrix.

		Predicted Condition	
		True	False
Actual condition	True	True Positive (TP)	False Positive (FP)
	False	False Negrative (FN)	True Negrative (TN)

**Table 3:** Numbers of training and test data set.

			Number of Training Data Set	Number of Test Data Set	Total
Type	Welding	Automatic	44,824	33,194	79,018
		Manual	168,790	190,285	359,075
	Tack welding		87,632	57,751	145,383
	Repair welding		89,732	45,504	149,549
Total			390,978	326,734	733,025

**Table 4:** Confusion matrix of the RF classification model.

		Predicted Result (RF)			
		Auto	Manual	Tack	Repair
Actual data	Auto	32,457	672	11	54
	Manual	2342	172,563	7833	7547
	Tack	10	11,350	42,356	4035
	Repair	58	8842	6400	30,204

**Table 5:** Confusion matrix of the ET classification model.

		Predicted Result (ET)			
		Auto	Manual	Tack	Repair
Actual data	Auto	32,515	609	12	58
	Manual	1756	17,4497	6878	7154
	Tack	21	11,612	42,604	3478
	Repair	66	9328	6798	29,312

**Table 6:** Confusion matrix of the GB classification model.

		Predicted Result (GB)			
		Auto	Manual	Tack	Repair
Actual data	Auto	32,319	791	17	67
	Manual	2790	168,210	9559	9726
	Tack	17	15,398	36,743	5593
	Repair	104	12,106	8185	25,109

**Table 7:** Confusion matrix of the ADA classification model.

		Predicted Result (ADA)			
		Auto	Manual	Tack	Repair
Actual data	Auto	32,858	227	18	91
	Manual	15,361	56,966	83,613	34,354
	Tack	57	4175	38,400	15,119
	Repair	964	2804	17,091	24,645

**Table 8:** Confusion matrix of the XGB classification model.

		Predicted Result (XGB)			
		Auto	Manual	Tack	Repair
Actual data	Auto	31,755	1282	10	147
	Manual	3432	168,895	10,449	7509
	Tack	13	19,277	34,642	3819
	Repair	116	17,563	11,908	15,914

**Table 9:** Confusion matrix of the STK classification model.

		Predicted Result (STK)			
		Auto	Manual	Tack	Repair
Actual data	Auto	32,506	628	13	47
	Manual	1746	174,821	6787	6931
	Tack	21	11,870	42,615	3245
	Repair	65	9269	6990	29,180

**Table 10:** Model performance by classifier.

Model	Weld Type	Performance Index			
		Precision	Recall	*F*1 Score	Accuracy (%)
RF	Auto	0.93	0.98	0.95	84.96
	Manual	0.89	0.91	0.90	
	Tack	0.75	0.73	0.74	
	Repair	0.72	0.66	0.69	
ET	Auto	0.95	0.98	0.96	85.38
	Manual	0.89	0.92	0.90	
	Tack	0.76	0.74	0.75	
	Repair	0.73	0.64	0.68	
GB	Auto	0.92	0.97	0.94	80.30
	Manual	0.86	0.88	0.87	
	Tack	0.67	0.64	0.65	
	Repair	0.62	0.55	0.58	
ADA	Auto	0.67	0.99	0.80	46.79
	Manual	0.89	0.30	0.45	
	Tack	0.28	0.66	0.39	
	Repair	0.33	0.54	0.41	
XGB	Auto	0.90	0.96	0.93	76.88
	Manual	0.82	0.89	0.85	
	Tack	0.61	0.60	0.60	
	Repair	0.58	0.35	0.44	
STK	Auto	0.95	0.98	0.96	85.43
	Manual	0.89	0.92	0.90	
	Tack	0.76	0.74	0.75	
	Repair	0.74	0.64	0.69	
